# Associations Between Children’s Media Use and Language and Literacy Skills

**DOI:** 10.3389/fpsyg.2020.01734

**Published:** 2020-08-05

**Authors:** Rebecca A. Dore, Jessica Logan, Tzu-Jung Lin, Kelly M. Purtell, Laura M. Justice

**Affiliations:** ^1^Crane Center for Early Childhood Research and Policy, The Ohio State University, Columbus, OH, United States; ^2^Department of Educational Studies, The Ohio State University, Columbus, OH, United States; ^3^Department of Human Sciences, The Ohio State University, Columbus, OH, United States

**Keywords:** media, language, literacy, screen time, children

## Abstract

Media use is a pervasive aspect of children’s home experiences but is often not considered in studies of the home learning environment. Media use could be detrimental to children’s language and literacy skills because it may displace other literacy-enhancing activities like shared reading and decrease the quantity and quality of caregiver–child interaction. Thus, the current study asked whether media use is associated with gains in children’s language and literacy skills both at a single time point and across a school year and whether age moderates any association. Children (*N* = 1583) were from preschool through third grade classrooms and language and literacy skills were measured in the fall and spring of the school year. Parents reported how much time their child spends using media on a typical school day. Regression analyses showed that using 4 h or more of media was related to lower literacy gains, but not to language gains. Multilevel models conducted as a robustness check showed that this effect did not hold when accounting for classroom. In neither set of models was there an interaction between age and media use. Single-time-point models did show some associations that did not manifest in more stringent models, highlighting the limitations of correlational designs that do not have measures of children’s skills over time. Given the concern and popular press coverage around children’s media use, it is important to acknowledge non-significant effects in this domain. These non-significant associations suggest that societal fears around children’s media use may be exaggerated. Notably, however, characteristics of children’s media use, like educational content or adult co-use, may moderate any effects. The relation between media use and language and literacy growth did not differ across the age range investigated suggesting that, within this range, younger children are not more vulnerable to detrimental effects.

## Introduction

Social-constructivist theories assert that children’s knowledge and skills develop in the context of interactions with others who have more experience, such as parents, caregivers, and other adults (Bruner, 1978). In line with this theoretical tradition, research has shown that the home learning environment, which captures both materials and opportunities for interactions, is linked to child development and learning across domains, including language and literacy (NICHD Early Child Care Research Network, 1999; Barnett et al., 2012; Hirsh-Pasek et al., 2015). Previous research has sought to understand how specific aspects of children’s experiences at home relate to language and literacy skills. For example, the number of picture books in the home and the frequency of parent–child shared reading are related to preschoolers’ language ability (Payne et al., 1994), as is time spent engaging in other educational or enriching activities like talking to adults (Fiorini and Keane, 2014).

However, media use is an increasingly pervasive activity in children’s lives in the 21st century, but it is often not considered in the context of research on the home environment. Children under 8 spend over 2 h a day with media and time spent using mobile devices tripled from 2013 to 2017 (Rideout, 2017). Indeed, children spend more time using media than in any other single leisure activity (Bianchi et al., 2006) and recent evidence suggests that early childhood technology use has increased by 32% in the last two decades (Goode et al., 2019).

Although the frequency of children’s media use is well established, its association with language outcomes is not as well understood. However, some research suggests that media use may be detrimental to children’s language and literacy skills because it may displace other literacy-enhancing activities like shared reading and decrease the quantity and quality of caregiver–child interaction. For example, Khan et al. (2017) found that higher levels of television viewing were associated with lower levels of parent–child book reading among a nationally representative sample of 4-year-olds. Furthermore, both the quantity and quality of caregiver speech and caregiver–child engagement is lower during television viewing compared with free play or other activities (Nathanson and Rasmussen, 2011; Pempek et al., 2011; Lavigne et al., 2015). This is critical because early caregiver–child interactions in the home are highly influential to language development (e.g., Hirsh-Pasek et al., 2015).

In line with these data, several studies have found that media exposure during toddlerhood or preschool is associated with lower language development in subsequent years (Clarke and Kurtz-Costes, 1997; Duch et al., 2013; Pagani et al., 2013). Furthermore, one recent study shows an association between children’s screen media use and lower microstructural integrity of brain white matter in areas related to language and emergent literacy (Hutton et al., 2019). However, findings are inconsistent, with other studies finding no association between media exposure and children’s language development (Patterson, 2002; Taylor et al., 2017; Pence and Alamos, 2019).

Notably, societal and parental concern about potential effects of media use on child development run counter to research showing that media can have some benefits for children. A large body of research shows that by preschool, children can learn from high-quality educational media, including in the domain of language and early literacy skills (e.g., Penuel et al., 2012; Mares and Pan, 2013; Hurwitz, 2018; Dore et al., 2019). There is also evidence that active video games or exergames, like *Wii Fit* or *Just Dance*, can increase children’s physical activity and fitness (e.g., Flynn et al., 2018; Gao et al., 2018). Although research is in its early stages, open-ended, multi-player games like *Minecraft* may also have the potential to promote creativity and collaboration (Lane and Yi, 2017). In line with these ideas, most parents believe that their child benefits from media use, especially in the areas of learning and creativity (Rideout, 2017). Thus, to the extent that families limit media because of concerns about effects on child development, including language and literacy, children may be missing out on positive aspects of media use.

Importantly, much of the literature focusing on the role of media use in language development has focused on infants, toddlers, and preschoolers, with less focus on older children. The preschool years are a time of immense language growth, as there are large increases in neural connections in the prefrontal cortex during this period that are influenced by early experiences (Huttenlocher, 2002). Thus, one might predict that media use would be more detrimental in preschool than during the elementary years. However, parents of 5- to 8-year-olds are even more likely than parents of younger children to believe that their child spends too much time with screen media and are less likely to believe that media use helps their child’s learning (Rideout, 2017), indicating that concerns about screen media use are still prevalent for this age group. Examining the relation between media use and language and literacy development with older children and across a wider age range is important to understand whether any relation is consistent across childhood or whether early childhood is an especially vulnerable time period for possible disruptions to the development of language and literacy skills. Investigating this question in the understudied age range used here (PreK to Grade 3) is particularly important to provide developmentally specific recommendations for parents and caregivers about children’s screen media use. We expect that there will be a stronger association between screen media use and language and literacy skills during early childhood, given rapid growth in these domains during the preschool years.

Another limitation of the existing research is that it focuses largely on vocabulary as an outcome to the exclusion of more comprehensive measures of language and literacy development. Although vocabulary is a vital aspect of language development and is predictive of later reading ability (Cunningham and Stanovich, 1997; Kim, 2016, 2017), letter knowledge and decoding are also necessary components of reading (e.g., Hoover and Gough, 1990; Chen and Vellutino, 1997; Kendeou et al., 2009) and media use may affect these skills via different mechanisms. Theoretically, whereas vocabulary may be affected if media leads to reduced opportunities for adult–child interaction and conversation, literacy skills are more likely to be affected if media displaces shared book reading and other print-focused activities. Thus, it is vital for research to examine multiple aspects of language and literacy development in relation to children’s media use to determine whether there are differential relations between media use and these skills.

Finally, many previous studies have examined the correlation between media use and language skills at one time point (e.g., Clarke and Kurtz-Costes, 1997) or have only an earlier measure of media use and a later measure of language skills (Pagani et al., 2013). Although experimental designs are necessary to draw strong conclusions about causality, using a design with measures of children’s language skills at two time points can begin to further our understanding of potential directionality of influence by controlling for initial levels of language and literacy skills and examining the unique relation between media use and change in these skills over time. Notably, we conduct analyses using both a single time point (i.e., analysis of correlation) and using two time points (i.e., analysis of pre–post changes) to test the extent to which media use was more associated with children’s language and literacy skills at the end of the academic year or the growth of these skills across the academic year.

Thus, the current study addresses four research questions: (1) To what extent is media use associated with gains in the language skills of children in PreK to 3rd grade across the school year? (2) To what extent is media use associated with gains in the literacy skills of children in PreK to 3rd grade across a school year? (3) Does age moderate any association between media use and language and literacy skills? (4) To what extent do the results of models assessing skill gains differ from single-time-point models?

## Materials and Methods

### Participants

Children from preschool through 3rd grade were recruited via their classroom teachers in two school districts in a Midwestern City in the United States. Every student within each classroom was asked to participate and 66.0% consented. Of the children whose parents consented for them to participate, approximately 74% of families returned the survey that included the media questions to be included in the current analysis. Of those, approximately 77% had data for the variables of interest for the current research questions. Thus, data from 1583 children (PreK *n* = 238, kindergarten *n* = 466, grade 1 *n* = 307, grade 2 = 326, and grade 3 = 246; 49.7% males) are included. Note that the sample size varies slightly for each analysis as a result of variable missingness across assessments, and so on. See Table 1 for sample demographics.

**TABLE 1:** Descriptive statistics for all study variables.

Continuous variables	Mean	*SD*
WJ picture vocabulary (Fall)	478.0	14.4
WJ picture vocabulary (Spring)	482.0	14.4
WJ letter word identification (Fall)	416.8	62.4
WJ letter word identification (Spring)	440.0	62.4

**Factors**	**Percentage**	

**Media use on a typical school day**		
0–1 h	39.9%	
2–3 h	53.4%	
4 h or more	6.8%	
**Mother’s education**		
Less than high school diploma	6.6%	
High school diploma or GED	27.2%	
Associate’s degree	12.4%	
Bachelor’s degree	29.6%	
Master’s degree	20.5%	
Doctoral degree	3.8%	
**Number of adults in the home**		
One	8.7%	
Two	79.5%	
More than two	11.3%	
**Child’s race**		
White	75.0%	
Hispanic or Latino	7.8%	
Black or African–American	4.5%	
Asian	5.9%	
American Indian or Alaska Native	0.02%	
Multiple races/ethnicities	10.8%	

### Procedures

Children’s language and literacy skills were directly assessed in the fall and the spring of the academic year. In the spring, caregivers reported on children’s media use, as well as other child and family demographic characteristics.

### Measures

#### Caregiver Report of Child Media Use

As part of a larger family background questionnaire, parents responded to two binary items asking whether their child watched “any kind of video, including TV, movies or short clips on any type of device” and played “games on any type of electronic device” on a typical school day. If parents reported that their child used any media on a typical school day, they were then asked how much time their children spends using media from five response options: 0–1, 2–3, 4–5, 6–7, and 8+ h. For analyses, the last three categories were combined into one category for 4 h or more, given small cell sizes.

#### Language Skills

To assess language skills, children completed the Picture Vocabulary subtest of the *Woodcock Johnson Test of Achievement-III* (WJ-III; Woodcock et al., 2007) in the fall and the spring of the school year. The initial items of the subtest require children to choose the picture that fits the named word for the initial items and then later items require children to provide names for each picture (44 items total). Six consecutive correct items are needed to establish test basal and six consecutive incorrect responses terminate the test. Reliability was adequate (0.80) and *W*-scores were used to examine student growth.

#### Literacy Skills

To assess literacy skills, children completed the Letter-Word Identification subtest of the *Woodcock Johnson Test of Achievement-III* (WJ-III; Woodcock et al., 2007) in the fall and the spring of the school year. This subtest (76 items total) requires children to identify individual letters and then read individual words of increasing difficulty. Six consecutive correct items are needed to establish test basal and six consecutive incorrect responses terminate the test. Reliability was adequate (0.94) and *W*-scores were used to examine student growth.

#### Demographic Characteristics

Several background demographic characteristics were assessed and included as control variables in our analyses. Specifically, age, gender, race, mother’s education, and number of adults in the household were reported by the caregiver as part of the larger family background questionnaire.

### Analysis Plan

We first report descriptive statistics and assess demographic characteristics as predictors of media use to understand the characteristics of children’s media use in the sample. Next, to assess the first two research questions, whether media use was related to gains in children’s language and literacy skills in early and middle childhood, we conduct regression analyses separately for language skills and literacy skills. Children’s spring *W*-scores are dependent variables and models control for fall scores to assess change. The models also control for age, gender, race, mother’s education, and number of adults in the household. Pursuant to the third research question, whether the relation between media use and children’s language development was moderated by age, we add an interaction between age and media use into the previously described models by multiplying the age and media use variables and examining whether the interaction variable is statistically significant predictor of children’s spring *W*-scores. Then, although our research questions are at the child level given that recruitment was conducted in classrooms and ICCs indicate that the grade and classroom accounted for a significant portion of the variability in the outcomes of interest (language ICC = 0.56; literacy ICC = 0.84), as a robustness check for our initial models we also conduct multilevel models accounting for classroom, testing for both main effects of media use and interactions with age. Finally, to be able to provide comparable results with previous studies using a single time point, we conduct separate regression analyses predicting children’s spring language and literacy scores from media use, also reported in the spring.

## Results

### Descriptive Statistics and Predictors of Children’s Media Use

**FIGURE 1:**
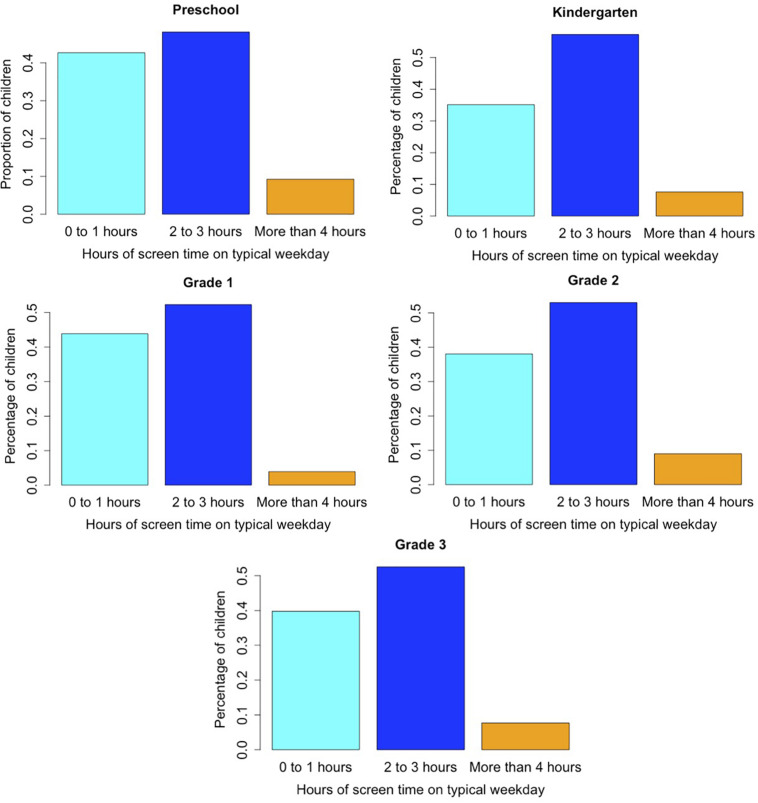
Parent report of children’s media use on a typical school day, by grade.

We first report descriptive statistics related to children’s media use. See Table 1 for descriptive statistics for all study variables. Parents reported that 71.5% of children both watch videos and play games on a typical school day, with an additional 22.2% watching video but not playing games and 1.4% playing games but not watching video. Only 4.8% of children were reported to not use any media on a typical school day. Of those children who were reported to use media on a typical school day, 39.4% use media for 0–1 h, 53.0% use media for 2–3 h, and 7.6% use media for 4 h or more (see Figure 1).

Children’s media use was related to mother’s education (*p*s < 0.0003), with children of mothers with lower education more likely to use 2–3 h or 4 h or more of media on a typical school day. Media use was also related to race (*p* = 0.04), such that White children were more likely to use 2–3 h of media on a typical school day (54%) than 0–1 h (38%), whereas non-White children were more evenly split between these categories (47 and 43%, respectively). Similarly, boys were more likely to use 2–3 h of media on a typical school day (56%) than 0–1 h (35%), whereas girls were more evenly split between these categories (49 and 44%, respectively). Children with a larger number of adults in the home were more likely to use 2–3 h of media on a typical school day than to use 0–1 h, *p* = 0.04. Media use was not related to child age, *p*s > 0.40.

### Relation Between Media Use and Language and Literacy Gains

Results of a model predicting children’s language gains showed that there was no association between media use and language gains, *p*s > 0.11, see Table 2. The interaction was not significant, *p*s > 0.21.

**TABLE 2:** Predicting language gains: results of a regression model (*N* = 1574).

Predictor	*B*	β	*SE*	*p*
Intercept	156.33		6.87	< 0.0001***
Baseline language	0.65	0.70	0.02	< 0.0001***
Media use (2–3 h)	–0.54	–0.02	0.34	0.12
Media use (more than 4 h)	0.06	0.001	0.66	0.93
Gender	–0.62	–0.02	0.32	0.06^+^
Age	0.15	0.20	0.01	< 0.0001***
Race (white)	1.80	0.05	0.47	0.0001**
Mother’s education	0.83	0.08	0.13	< 0.0001***
Number of adults in household	0.30	0.01	0.27	0.27
			Adjusted *R*^2^ = 0.769	

Outcome is Picture Vocabulary W-scores. Media use reference group is “0–1 h.” The interaction between media use and age was not significant and was removed from the model. ^+^*p* < 0.10, **p < 0.001 and ***p < 0.0001.

**FIGURE 2:**
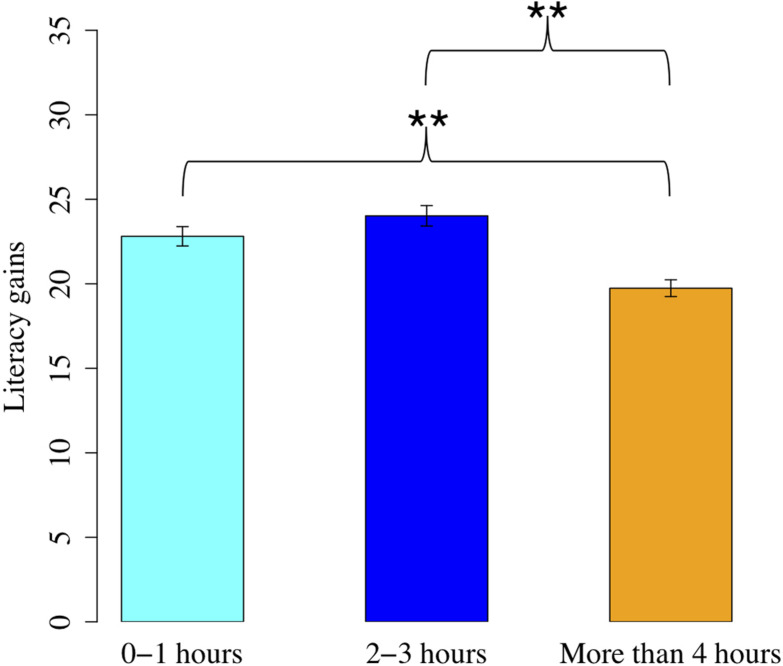
Association between media use and literacy gains. **p < 0.001.

In the model for literacy, results showed that children who used 4 or more hours of media on a typical school day had significantly smaller literacy gains than children who used 0–1 h of media per day (*B* = 5.2, *p* = 0.002) or those who used 2–3 h of media per day (*B* = 4.7, *p* = 0.005; see Table 3 and Figure 2). Using media 2–3 h per day was not associated with smaller gains than 0–1 h per day, *p* = 0.58. As aforementioned, we added an interaction between age and media use into the previously described model to test our third research question. There was no interaction between age and screen media, *p*s > 0.21.

**TABLE 3:** Predicting literacy gains: results of a regression model (*N* = 1582).

Predictor	*B*	β	*SE*	*p*
Intercept	75.99		3.41	< 0.0001***
Baseline literacy	0.83	0.91	0.01	< 0.0001***
Media use (2–3 h)	–0.48	–0.004	0.86	0.58
Media use (more than 4 h)	–5.17	–0.02	1.70	0.002**
Gender	0.45	0.004	0.82	0.59
Age	0.19	0.06	0.05	< 0.0001***
Race (white)	–0.46	–0.003	1.15	0.69
Mother’s education	–0.88	–0.02	0.33	0.008**
Number of adults in household	1.45	0.02	0.69	0.04*
			Adjusted *R*^2^ = 0.920	

Outcome is Letter Word W-scores. Media use reference group is “0–1 h.” *p < 0.05, **p < 0.01 and ***p < 0.001.

In the multilevel robustness check model for language, as in the initial model, there was no association between media use and language gains, *p*s > 0.16, see Table 4. The interaction between age and media use was also not significant, *p*s > 0.27.

**TABLE 4:** Predicting language gains: results of multilevel regression model (*N* = 1570).

Predictor	*B*	β	*SE*	*p*
Intercept	155.60		6.95	< 0.0001***
Baseline language	0.65	0.70	0.02	< 0.0001***
Media use (2–3 h)	–0.48	–0.02	0.34	0.16
Media use (more than 4 h)	0.23	0.005	0.66	0.72
Gender	–0.61	–0.02	0.32	0.06^+^
Age	0.15	0.19	0.01	< 0.0001***
Race (white)	1.81	0.05	0.48	0.0002**
Mother’s education	0.83	0.08	0.13	< 0.0001***
Number of adults in household	0.31	0.01	0.27	0.26
			Adjusted *R*^2^ = 0.776	

Outcome is Picture Vocabulary W-scores. Media use reference group is “0–1 h.” The interaction between media use and age was not significant and was removed from the model. ^+^*p* < 0.05, **p < 0.001 and *** *p* < 0.0001.

Unlike the initial model, the association between media use and literacy gains was not significant in the multilevel robustness check model, *p*s > 0.10, see Table 5. We expect that this difference is a result of reduced power in the more stringent model and of the effects of classroom on children’s literacy skills that wash out smaller associations with home media use. The interaction between age and media use was also not significant, *p*s > 0.54.

**TABLE 5:** Predicting literacy gains: results of multilevel regression model (*N* = 1578).

Predictor	*B*	β	*SE*	*p*
Intercept	76.71		4.66	< 0.0001***
Baseline literacy	0.83	0.91	0.01	< 0.0001***
Media use (2–3 h)	–0.35	–0.003	0.75	0.64
Media use (more than 4 h)	–2.42	–0.01	1.49	0.10
Gender	0.59	0.01	0.70	0.40
Age	0.18	0.05	0.05	0.0008**
Race (white)	–1.46	–0.01	1.05	0.16
Mother’s education	0.04	0.001	0.33	0.91
Number of adults in household	0.88	0.01	0.60	0.15
			Adjusted *R*^2^ = 0.946	

Outcome is Letter Word W-scores. Media use reference group is “0–1 h.” The interaction between media use and age was not significant and was removed from the model. **p < 0.001 and ***p < 0.0001.

### Relation Between Media Use and Language and Literacy Skills at a Single Time Point

To address our fourth research question, about the extent to which the results of models assessing skill gains differ from single-time-point models, we examined the association between media use and language skills at a single time point. Results showed a significant interaction (*B* = −0.16, *p* = 0.004; see Table 6). Follow-up regression analyses for each age quartile showed that there was only a negative association between media use and language skills for children in the oldest quartile, 99 months (or a little over 8 years) and older, among whom having more than 4 h a day of media use per day was associated with lower language skills than having 0–1 h of media use (*B* = −4.36, *p* = 0.03), whereas there was no significant association between media use and language skills for younger children, *p*s > 0.13.

**TABLE 6:** Predicting spring language scores: results of a regression model (*N* = 1583).

Predictor	*B*	β	*SE*	*p*
Intercept	423.91		2.12	< 0.0001***
Media use (2–3 h)	3.50	0.13	2.47	0.16
Media use (more than 4 h)	13.53	0.25	4.75	0.004**
Gender	–0.50	–0.02	–1.09	0.28
Age	0.54	0.69	0.02	< 0.0001***
Race (white)	7.10	0.19	0.65	0.0001**
Mother’s education	2.33	0.24	0.17	< 0.0001***
Number of adults in household	0.32	0.01	0.39	0.41
Media use (2–3 h) × age	–0.05	–0.16	0.03	0.09^+^
Media use (more than 4 h) × age	–0.16	–0.26	0.05	0.004**
			Adjusted *R*^2^ = 0.534***	

Outcome is Picture Vocabulary W-scores. Media use reference group is “0–1 h.” ^+^*p* < 0.05, **p < 0.01 and ***p < 0.0001.

When examining the association between media use and literacy skills at a single time point, results showed a significant main effect of media use, such that using 2–3 h of media per day was associated with lower literacy scores than using 0–1 h of media use (*B* = −6.00, *p* = 0.0002) and using more than 4 h of media per day was associated with lower literacy scores than using either 0 to hours (*B* = −12.01, *p* = 0.0001) or 2–3 h (*B* = 6.01, *p* = 0.049; see Table 7). The interaction between media use and age was not significant.^1^

**TABLE 7:** Predicting spring literacy scores: results of a regression model (*N* = 1583).

Predictor	*B*	β	*SE*	*p*
Intercept	191.53		5.23	< 0.0001***
Media use (2–3 h)	–6.00	–0.05	1.59	0.0002**
Media use (more than 4 h)	–12.01	–0.05	3.12	0.0001**
Gender	2.29	0.02	1.5	0.13
Age	2.78	0.82	0.04	< 0.0001***
Race (white)	–2.18	–0.01	2.11	0.30
Mother’s education	6.53	0.15	0.57	< 0.0001***
Number of adults in household	0.49	0.01	1.27	0.70
			Adjusted *R*^2^ = 0.728	

Outcome is Letter Word W-scores. Media use reference group is “0–1 h.” The interaction between media use and age was not significant and was removed from the model. **p < 0.001 and ***p < 0.0001.

## Discussion

This investigation was motivated by mixed findings in previous research, with some studies showing that media use was related to lower language development and others showing no relation. The current study had several strengths, including a large sample and data on children’s language and literacy skills collected at two time points. In our initial gain models, we found that only high levels of media use were related to smaller gains in skills and only for literacy, not for language. Furthermore, a robustness check indicated that even this effect was not strong enough to emerge in a model that accounted for the nested structure of the data. However, in single-time-point models, we found main effects of media use on children’s literacy skills and an interaction between media use and age predicting language skills, such that there was an association only for the older children in our sample.

The discrepancy between the single-time-point and gain models highlights the limitations of correlational designs that do not have measures of children’s skills over time. If we were relying on a single time point, we would draw markedly different conclusions from these data. If children’s media use has a detrimental effect on language and literacy skills, we would expect it to continue to operate and manifest in gains over time. Instead, we find that the effects in the single-time-point models are not significant in the most robust gain models. This suggests that perhaps there is a third variable problem where media use is acting as a proxy for other family or home characteristics that are also associated with children’s language and literacy skills. Accounting for fall scores largely eliminates these associations, leaving only the unique association between media use and language and literacy gains.

Given the concern and popular press coverage around children’s media use, it is important to acknowledge non-significant effects in this domain. There has been fear around the effects of novel technologies and mediums in the past, including concerns about the effects of exposure to early radio shows in the 1920s and moral panic about children reading comic books in the 1950s (Wartella and Jennings, 2000; Tilley, 2012). Although children’s media use in the 21st century may appear extreme and strikingly different from the childhood their parents experienced 30 or 40 years ago, non-significant associations such as those reported here suggest that societal fears around children’s media use may be exaggerated.

Importantly, even in our initial gain models, we only found associations between media use and literacy gains for high levels of use, whereas literacy gains did not differ between more moderate levels of use and low use. These data suggest that any effect of media use on literacy skills may represent a threshold effect, rather than a linear relationship. In addition to the use of single-time-point approaches, this type of relation may explain mixed findings in the literature because analyses testing only for a linear relationship may not be sensitive enough to detect this kind of threshold. Why might this threshold effect exist? One possible explanation is that media use has a negative effect on literacy skills when it is used in large amounts on a daily basis and thus displaces important educational activities like shared book reading and other activities with a print focus. On the other hand, more moderate amounts of media use may be less likely to displace such activities or may not displace them to the extent that it disrupts literacy development. The somewhat small proportion of children falling into the highest category of media use (7.6% using media for 4 or more hours on a typical school day) may contribute to lower power to detect effects of this extreme amount of use in our robustness check models.

Given previous findings suggesting that media use can displace important language- and literacy-enhancing activities, the non-significant effects reported here are somewhat surprising. However, there are several possible explanations for these findings. Importantly, media use is not monolithic, and some media experiences could be more supportive of language development than others (Linebarger and Vaala, 2010; Dore et al., 2017). We measured only quantity of media use but features of that media use may moderate any effects on language development. First, the relation between media and language development may depend on how much adults engage with children during media use. In other words, the role that media use plays in a child’s language and literacy development may differ depending on whether she primarily uses media by herself or whether most of her media use involves a caregiver using and discussing media with her. When this type of joint media engagement is frequent, language and literacy development may be more positive because the media experience does not replace caregiver–child interaction and instead extends it to a new context. Second, given the importance of joint attention and serve-and-return responding to language development (e.g., Tomasello and Farrar, 1986), it may be significant that some types of media are themselves interactive. That is, digital games and apps can be responsive to the child’s actions in a way that a television show is not (Sheehan and Uttal, 2016). Although the interactivity afforded by digital devices is less flexible and responsive than a caregiver might be, the proportion of children’s media use that includes interactive rather than non-interactive media may differentially predict language and literacy skills because children are more likely to be actively engaged when media uses interactive features. Third, educational media may be more supportive of language and literacy development than content that is intended primarily for entertainment, as research suggests that children can learn from high-quality television (Mares and Pan, 2013). Because most previous research and the current study focus solely on quantity of media use and do not allow for an examination of these factors, null effects and conflicting findings may mask important effects that would emerge when these moderators are considered.

Another possible contributor to these findings relates to the age range of our sample. Most previous research that has found negative relations between media use and language development has been conducted with younger children. Media use may not displace the same activities for older children as it does for toddlers and younger preschoolers. For younger children, media use may most commonly displace activities like storybook reading and parent–child interaction, whereas for older children who are becoming more independent, it may be more likely to displace peer and independent play which are likely to be less important for language and literacy development. In line with this idea, a recent study showed that media use was associated with lower physical activity among 6- to 11-year-olds but not with time for unstructured play (Goode et al., 2019).

An important contribution of the current study is that we used a wide age range, including older children than have typically been studied in past research in this domain. Many of the previous studies have focused on infants and toddlers, and little research has investigated the relation between media use and language and literacy for children in the early elementary grades. Our findings show that the inverse relation between these variables may not extend to this older age range, perhaps because early childhood is a more sensitive time for language development. Notably, we also found that the relation between media use and language and literacy growth did not differ across the age range investigated (PreK to 3rd grade), suggesting that any effects in early childhood may be restricted to toddlers and the early preschool years.

Although we find non-significant relations between media use and language and literacy skills in our most robust models, there may be detrimental effects of media use in other domains. For example, children’s media use, and evening media in particular, has been associated with increased sleep problems and changes in sleep patterns (Garrison et al., 2011; Beyens and Nathanson, 2019). Some studies also find that preschoolers’ media use is correlated with lower social skills (Hinkley et al., 2018) and executive function skills (Nathanson et al., 2014). It will be important for future research to investigate the relation between media use and other domains of child development. Furthermore, although we had a large and somewhat diverse sample, we did not have data for all of the children in our sample because of missing surveys and items, and our findings may not generalize to other populations. Future research should investigate these relations in a representative sample of children and families.

Although additional research is needed to further elucidate the role that media plays in child development, the current findings suggests that, at least by PreK and for language and literacy skills, any role of media use in children’s development is likely to be small at most. There may be important moderators that were not examined here, such that certain types of media use or media use in certain contexts may be more strongly associated with children’s skills. Importantly, children may also benefit from some media use, such as video games and exergames (Blumberg et al., 2019), and overgeneralizations that demonize media use could lead to children missing out on valuable opportunities for media to contribute to positive aspects of their lives. Given its prevalence in children’s lives, media use is a fundamental aspect of the home environment and it will be important for future research to investigate how it relates to both other aspects of the home environment and to children’s developing skills.

## Data Availability Statement

The raw data supporting the conclusions of this article will be made available by the authors, without undue reservation, to any qualified researcher.

## Ethics Statement

The studies involving human participants were reviewed and approved by The Ohio State University Institutional Review Board. Written informed consent to participate in this study was provided by the participants’ legal guardian/next of kin.

## Author Contributions

LJ, KP, T-JL, and JL contributed to conceptualization of the larger project and obtained funding. RD and JL contributed to the conceptualization of the current study and conducted data analyses. RD drafted the work. JL, LJ, KP, and T-JL contributed to editing and revising and gave final approval of the version to be submitted. All authors contributed to the article and approved the submitted version.

## Disclaimer

The opinions expressed are those of the authors and do not represent views of the Institute or National Center for Education Research.

## Conflict of Interest

The authors declare that the research was conducted in the absence of any commercial or financial relationships that could be construed as a potential conflict of interest.

## Footnotes

1 These models were also conducted as multilevel models accounting for classrooms and results were similar.
